# The feasibility of using pathobiome strains as live biotherapeutic products for human use

**DOI:** 10.1002/imt2.202

**Published:** 2024-05-26

**Authors:** Pengfei Jin, Xiong Lin, Wenfeng Xu, Kangning Li, Xiaoxiao Zhao, Sirui Guo, Zinan Zhao, Lujie Jiang, Feng Liao, Longgang Chang, Min Wang, Yanmin Liu, Shaolei Huang, Zhangran Chen, Fusui Ji

**Affiliations:** ^1^ Department of Pharmacy Beijing Hospital, National Center of Gerontology; Institute of Geriatric Medicine, Chinese Academy of Medical Sciences; Beijing Key Laboratory of Assessment of Clinical Drugs Risk and Individual Application (Beijing Hospital) Beijing China; ^2^ Shenzhen Wedge Microbiology Research Co., Ltd. Shenzhen China; ^3^ Department of Cardiology, Beijing Hospital, National Center of Gerontology; Institute of Geriatric Medicine Chinese Academy of Medical Sciences Beijing China

## Abstract

The evaluation of pathobiome strains should be conducted at the strain level, involving the identification of the functional genes, while considering the impact of ecological niche and drug interactions. The safety, efficacy, and quality management of live biotherapeutic products (LBPs), especially pathobiome strains, have certain peculiarities. Promising development methods include the recombinant LBP and active metabolites.

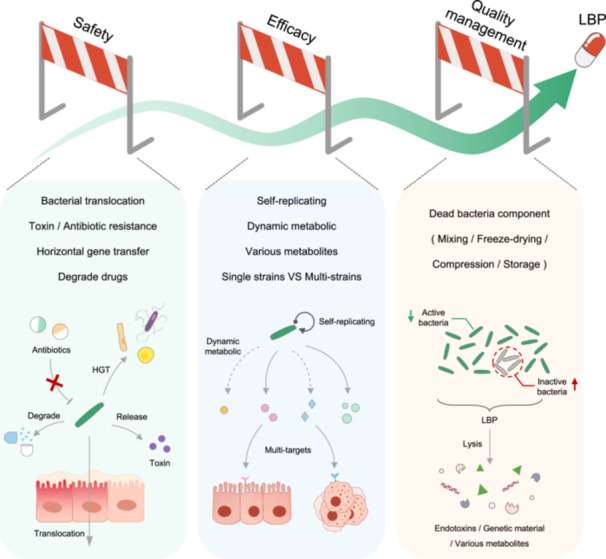

As understanding of the gut microbiota's role in human health deepens, it is increasingly recognized that the gut microbiota is not only related to the gastrointestinal tract but also closely associated with various organs and diseases in the human body. In addition to well‐known probiotics such as *Lactobacillus* and *Bifidobacterium*, the pathobiome, like *Escherichia*, *Bacteroides*, and *Enterococcus*, also plays important roles. The term “pathobiome” originates from human microbiome research, which may interact with the host and potentially reduce host health status [1]. How to assess the clinical pathogenicity and potential benefits of pathobiome? Can they be transformed into live biotherapeutic products (LBPs) to exert beneficial effects?

## IS PATHOBIOME HARMFUL OR BENEFICIAL TO HUMAN HEALTH?

First, addressing this question requires recognizing the common individual variations among different strains of the same bacterial species. Even through the construction of pan‐genomes for specific bacterial species to assess their properties, it is challenging to accurately classify the goodness or badness of the species. For instance, pathogenic *Escherichia coli* can cause various diseases such as sepsis and meningitis, whereas the complex flora with *E. coli* as the cornerstone can effectively resist the colonization of *Klebsiella pneumoniae* through nutrient competition [2]. Among these, *E. coli* Nissle 1917 (EcN 1917) is widely used as an engineered chassis cell and drug delivery vector for exploring various human disease treatment methods [3]. *Bacteroides fragilis* is divided into enterotoxigenic *B. fragilis* strains (ETBF) and non‐toxigenic *B. fragilis* strains (NTBF). ETBF is usually considered an opportunistic pathogen, while NTBF may have beneficial effects. Therefore, scientific evaluation should be based on the strain level rather than the species level.

Furthermore, differences between strains primarily arise from specific functional genes within the genome, leading to variations in secretions and cellular structures, such as bacterial toxins, enzymes, extracellular polysaccharides, lipoproteins, and protein glycosylation. The presence or absence of these functional genes determines whether a strain has the potential to impact human health. For example, *Helicobacter pylori* strains carrying pathogenic genes like *cagA* and *vacA* are considered highly virulent. Similarly, only *E. coli* strains capable of expressing toxins such as colibactin, enterotoxin, or hemolysin show clinical pathogenicity. Moreover, one bacterial species may carry both beneficial and harmful functional genes. For instance, *Enterococcus faecalis* or *Enterococcus faecium* may harbor various bacteriocin genes that help clear multiple human pathogens, enhancing their ecological competitiveness. Additionally, studies have shown that *epx*‐carrying *E. faecalis* or *E. faecium* strains can express a pore‐forming toxin, which currently shows higher toxicity in human cells than perforins sourced from other pathogenic bacteria [4]. Similarly, this complexity also exists in *B. fragilis*. The toxin gene *bft* is a typical representative of harmful genes in *B. fragilis*, encoding a zinc‐dependent metalloprotease that disrupts the intestinal epithelial barrier by cleaving E‐cadherin on the basolateral surface of colonocytes. On the other hand, capsule polysaccharides A serve as the primary functional molecules contributing to beneficial effects, with extensive research indicating their potential to reduce abscesses, prevent colitis, and minimize bacterial infections. However, it is important to note that the same gene may show both beneficial and harmful effects. Different studies have found that capsular polysaccharide A can both activate CD4^+^ T cells to secrete IL‐10 to suppress inflammation and induce the secretion of IFNγ, TNF‐α, IL‐6, and CXCL10, thereby promoting inflammation [5]. Therefore, evaluating the superiority or inferiority of specific strains requires attention to the functional genes and regulatory mechanisms within the strain.

Next, it is essential to consider the regulation of strain‐specific functional gene expression by ecological niche environments, particularly the interference of symbiotic microbiota, which may result in different gene expression patterns for strains in different ecological niches, thereby playing dual roles of either benefit or harm. Infections with *E. faecalis* OG1RF increase the mortality rate of nematode hosts; however, when coinfected with highly pathogenic *Staphylococcus aureus*, *E. faecalis* OG1RF can increase the secretion of its own reactive oxygen species to inhibit the growth of *S. aureus*, depleting the iron carriers secreted by *S. aureus*, thereby reducing the toxicity of *S. aureus* to the nematode host [6]. Similarly, research has found that monoassociated IL‐10‐deficient mice develop chronic colitis when exposed solely to *E. faecalis*. However, in the presence of multiple bacterial species, the transcriptome profile of *E. faecalis* changes, showing preventive effects against colitis through ethanolamine metabolism [7]. Furthermore, *E. faecalis* can reshape the environment and enhance *Clostridium difficile* virulence by metabolically modulating arginine and ornithine levels [8]. Therefore, the evaluation of strain superiority or inferiority also depends on the ecological niche in which the strain resides and even its impact on other strains.

Lastly, the interaction between bacteria and drugs is also a factor to consider, as the gut microbiota can influence the efficacy, toxicity, and bioavailability of drugs. In vitro experiments analyzed the metabolic effects of 25 intestinal bacteria on 15 drugs, identifying 29 new interaction relationships. Certain bacteria can accumulate drugs internally by binding with duloxetine via metabolic enzymes, thereby reducing the drug's bioavailability in the nematode hosts [9]. Additionally, bacterial tyrosine decarboxylase can metabolize levodopa into dopamine, affecting the efficacy of Parkinson's medication [10].

**Figure 1:**
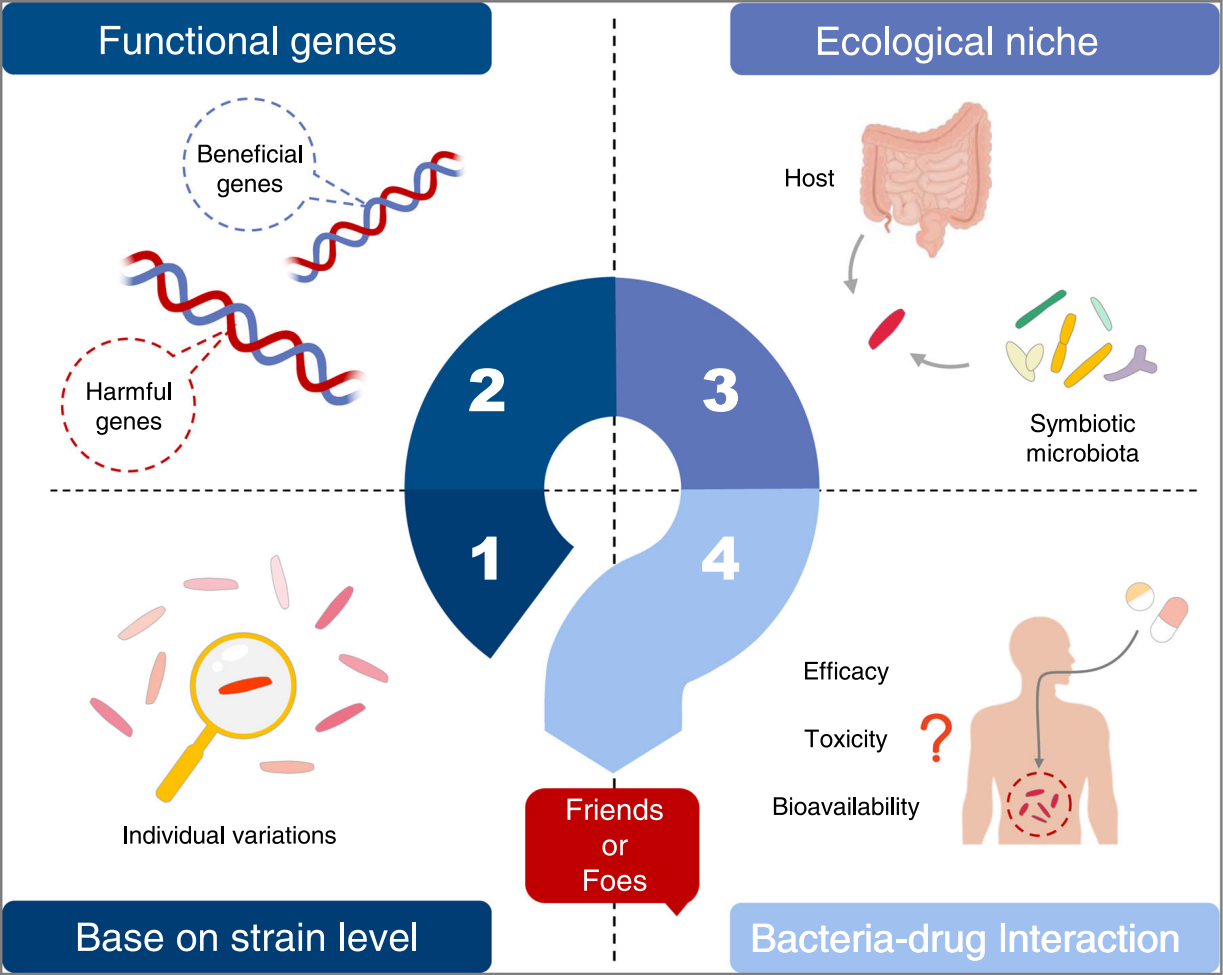
The four perspectives for evaluating the goodness or badness of pathobiome strains. Initially, evaluation should be conducted at the strain level. Subsequently, screening and identification of functional genes become imperative. Third, assessments should be executed within relevant ecological niches. Lastly, the ramifications of bacteria–drug interactions must be taken into account. Certain icons in the figure were drawn by Figdraw.

In summary, a scientific evaluation should be based on the strain level, focusing on functional genes within the strain as well as the metabolic patterns and gene expression regulation in the specific ecological niche. It is also important to consider drug metabolism issues to accurately assess their impact on human health (Figure 1).

## THE MAIN CHALLENGES IN UTILIZING PATHOBIOME STRAINS TO DEVELOP LIVE BIOTHERAPEUTIC PRODUCTS

**Figure 2:**
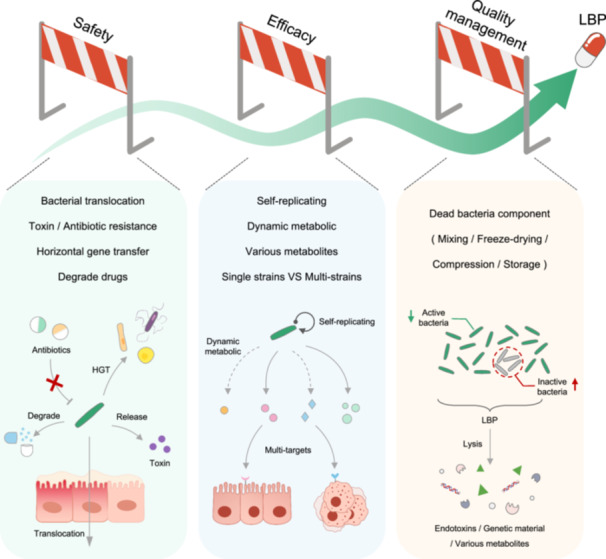
The main obstacles in developing pathobiome strains into live biotherapeutic products. Developing live biotherapeutic products using pathobiome strains requires consideration of safety, efficacy, and quality management. However, given their status as live organisms, they also possess their own uniqueness and complexity. HGT, horizontal gene transfer; LBP, live biotherapeutic product.

Currently, the variety of LBPs is relatively limited, with their indications primarily focused on gastrointestinal diseases. However, in reality, they have a long history of development and utilization, and the relevant bacterial fermentation industrial technologies and equipment are relatively mature. Nevertheless, as time progresses, the requirements for drug registration and approval continue to increase, demanding higher standards for drug safety, efficacy, and quality management (Figure 2). Moreover, LBPs, being live entities, entail a certain level of complexity and uniqueness in their drug development process, especially concerning pathobiomes.

The primary obstacle is concern regarding safety. First, bacterial infections caused by the translocation of pathogenic microorganisms in clinical settings are the most common issue, especially several common pathogens, including *E. faecium*, *S. aureus*, *K. pneumoniae*, *Acinetobacter baumannii*, and *Pseudomonas aeruginosa*, causing infections in the blood, surgical sites, urinary tract, and other body sites. Clinical cases have even reported that the enterococci in five infected premature infants were homologous to the enterococci found in probiotic medications administered in the hospital [11]. Even lactobacilli may cause bacterial infections in patients with severe immunodeficiency [12]. Second, the presence and transfer of antibiotic‐resistance genes and toxin‐producing genes in the pathobiome genome also pose safety risks. Although the horizontal transfer of antibiotic resistance genes between bacteria in vivo is still a scientific inference, numerous in vitro experiments have demonstrated varying frequencies of gene transfer between different bacterial species. For example, in hospital wastewater, it was found that the pX3_NDM‐5 plasmid containing the carbapenem‐resistant gene *bla*_NDM‐5_ could spread from *E. coli* to 16 different bacterial phyla [13]. The widespread existence of antibiotic‐resistance genes in bacteria means that strains with a certain level of antibiotic resistance can be used in combination with antibiotics to enhance efficacy or reduce side effects, suggesting potential therapeutic applications. The real vigilance comes from pathogens that tolerate salvage therapy antibiotics and pose risk of genetic transfer horizontally. Similarly, for toxin genes, what truly warrants vigilance are toxin genes that can be expressed in vivo and directly cause harm to the human body, rather than all virulence genes, because certain virulence factors associated with cell adhesion, colonization, and biofilm formation are related to the ecological niche competitiveness of target strains. These virulence factors are crucial for the beneficial effects exerted by the strains [14]. Furthermore, microbiomics has revealed significant differences between the gut microbiota of healthy individuals and patients, indicating correlation between specific bacterial species/genera and clinical diseases. Although the causation remains unclear, this may suggest the presence of new pathogenic factors. The third concern regarding safety relates to the target strains themselves or their impact on drug metabolism, as illustrated by the examples mentioned in the preceding section. Of course, intestinal bacteria may also play a beneficial role in drug metabolism. For example, *Lactobacillus vaginalis* secretes β‐galactosidase, which releases the isoflavone daidzein from common dietary sources, thereby alleviating acetaminophen‐induced hepatotoxicity in mice [15]. However, research on bacterial–drug interactions remains relatively limited thus far.

The second obstacle is complex drug efficacy. LBPs are capable of self‐replication, and the production of metabolites is dynamic. The mechanism of action of LBPs mainly involves regulating the gut microbiota through ecological competition and producing beneficial metabolites at certain concentrations. Therefore, the efficacy of LBPs is correlated with their quantity and metabolic intensity. However, bacterial metabolic intensity is influenced by the ecological niche environment, including host immune status, gastrointestinal damage, pH changes, and microbial interactions. Additionally, the diverse range of bacterial metabolites and the presence of multiple effective targets make efficacy research more complex. Furthermore, despite current research and development of live biotherapeutics predominantly focusing on single strains, there is also a perspective that multi‐strain formulations may be more suitable for addressing complex microbial environments, capable of inducing greater microbial perturbation. However, the development of multi‐strain formulations requires further investigation into the included species, doses, and interactions among the strains within the formulation. This undoubtedly adds complexity to preclinical studies and future manufacturing processes. Nonetheless, it is undeniable that the choice between single strains and multi‐strain formulations still hinges on their efficacy for the target indications.

The third obstacle is that product quality management is often neglected. During the production process of LBPs, such as mixing, freeze‐drying, and tablet compression, as well as with the increase in storage time on the shelf‐life, it is inevitable that the proportion of live bacteria in the product will gradually decrease, while the proportion of dead bacteria will gradually increase. However, existing quality management typically focuses only on the quantity of live bacteria while neglecting the impact of dead bacteria. Dead bacteria may still be effective or cause side effects, especially endotoxins released from bacterial lysis. Insufficient research on the efficacy and safety of dead bacteria may pose obstacles to the development of LBPs.

## POSSIBLE FUTURE RESEARCH DIRECTIONS AND APPLICATIONS

Based on the latest scientific research and challenges in industrial applications, we propose possible future research directions and application approaches. First, a deeper understanding of both the safety and efficacy of candidate therapeutic microbial strains requires further investigation into gene functions to uncover new functional genes, pathogenic factors, and regulatory mechanisms. This will allow us to analyze the functionality and safety of strains preliminarily through multi‐omics approaches such as whole‐genome sequencing, proteomics, and metabolomics. Second, attention should be paid to the influence of ecological niche, especially symbiotic microbiota on the gene expression of candidate strains. Establishing in vitro organ or animal models that allow coexistence with microbiota is necessary to further explore the gene expression patterns of specific strains and determine whether the concentration of effective metabolites is sufficient to impact human health, thus evaluating the therapeutic potential of the strains. Lastly, due to the insufficient research background at present, even nontoxic pathbiome strains should be studied with caution in preclinical research, focusing on their pathogenicity in specific disease animal models, and monitoring bacterial translocation and horizontal gene transfer in immunocompromised patients during clinical trials.

For indications of LBPs, beyond gastrointestinal diseases, considerations can extend to dermatological conditions, vaginal disorders, mental health disorders, and metabolic diseases. However, the scope of applications for live biotherapeutics is not limited solely to treating or alleviating diseases; equally promising scenarios involve reducing drug toxicity, side effects, or enhancing efficacy, as well as roles in disease prevention. For instance, indole‐3‐propionic acid produced by *Lactobacillus reuteri* from tryptophan metabolism can enhance the efficacy of anti‐programmed cell death protein 1 antibody immunotherapy, slowing tumor growth in mice [16]. Similarly, introducing the β‐lactamase gene into *Lactococcus lactis* can reduce levels of colon‐amoxicillin and protect gut microbiota, countering *C. difficile* infections post‐antibiotic treatment [17].

In terms of development methods, the most widely used strategy currently involves large‐scale screening to obtain nontoxic strains with potential medicinal value. In the future, gene editing technology may be considered to balance the safety and efficacy of LBP by knock‐in of beneficial functional genes and knockout of harmful ones, such as removing toxin genes or introducing tumor‐suppressing genes. Currently, considerable scientific research is being conducted on recombinant LBP, especially in the field of cancer treatment. For instance, genetically modified attenuated *P. aeruginosa* can target tumors under low oxygen conditions and release exotoxins to kill cancer cells [18]. Genetically modified EcN1917 targets tumors and releases L‐arginine, combined with anti‐PD‐L1, significantly inhibiting tumor growth [19], or targets the intestine and produces butyric, thereby mitigating inflammation [20]. Using engineered bacteria as drug carriers for in situ synthesis of therapeutic agents is particularly suitable for compounds with strong cytotoxicity, short half‐life, or relatively complex structures that are challenging to purify using traditional methods. For example, a novel antibacterial small molecule called epifadin was recently discovered in *Staphylococcus epidermidis*, which shows broad‐spectrum resistance and a short half‐life. It exerts potent bactericidal effects against *S. aureus* while having a minimal impact on the viability of normal human cells [21].

Furthermore, some biotechnology companies are investigating the potential indications for recombinant LBP, including various solid tumors, diabetic foot ulcers, psoriasis, the rare skin disorder Netherton syndrome, and phenylketonuria. Among these, cancer therapy and dermatological indications are currently the primary areas of focus. Most are in the preclinical exploration stage, while some have progressed to late‐stage clinical trials. However, despite the anticipation surrounding certain companies entering clinical trials, some have been forced to halt development due to the failure to meet expected endpoints in Phase III clinical trials. The approval of clinical trial ethics reviews provides significant encouragement to researchers. Nevertheless, it cannot be denied that there is still a long road ahead for recombinant LBP to transition from scientific research success to commercial success. The application of recombinant LBP faces challenges such as low public acceptance, unresolved policies, and the need for further safety testing, including potential off‐target effects of gene editing, how to prevent the escape of specific strains, and how to control the spread of specific genes.

In addition, the application of bacterial active metabolites is also a possible option, i.e., extracting large molecular active ingredients difficult to synthesize chemically through bacterial fermentation, such as extracellular polysaccharides, and even inactivated forms of bacteria, also known as postbiotics. The most widely known example would be the pasteurized *Akkermansia muciniphila*. The specific protein Amuc_1100 on its outer membrane remains active after pasteurization at 70°C and has the ability to reduce fat mass development, insulin resistance, and dyslipidemia in mice, and has been shown to be safe in obese individuals, with no adverse effects in preliminary human data. This is expected to be applied to improve obesity and other related diseases [22]. Of course, such active metabolites do not fall within the category of LBPs but align more with the development principles of modern drugs, being more direct, targeted, and dose‐controllable.

In conclusion, it is inappropriate to simply categorize pathobiome strains as either beneficial or detrimental. Each strain requires an extensive examination of its genetic functions and metabolic pathways, necessitating research into gene functionality, microbiota interactions, and the intricate interplay between strains and hosts. A profound comprehension of the strain's background lays the groundwork for potential transformation into LBPs. This transformation can be facilitated through various methods, including large‐scale strain screening, recombinant LBP, or the extraction of potent active metabolites. Despite the potential arduousness and complexity of such endeavors, the pursuit of such research avenues holds significant promise and warrants dedicated exploration and further investigation.

## AUTHOR CONTRIBUTIONS

Pengfei Jin conceived, wrote, and revised this manuscript. Xiong Lin wrote and revised this manuscript. Wenfeng Xu, Kangning Li, Xiaoxiao Zhao, Sirui Guo, Zinan Zhao, and Lujie Jiang conducted data collection and gave conceptual advice for the manuscript. Feng Liao, Longgang Chang, Min Wang, Yanmin Liu, and Shaolei Huang participated in discussions and gave conceptual advice for the manuscript. Zhangran Chen conceived, revised, and coordinated this work. Fusui Ji supervised this work and gave conceptual advice related to this work. All authors have read the final manuscript and provided approval for publication.

## CONFLICT OF INTEREST STATEMENT

The authors declare no conflicts of interest.

## ETHICS STATEMENT

No animals or humans were involved in this study.

## Data Availability

No new data were generated in this study. Supplementary materials (graphical abstract, slides, videos, Chinese translated version, and update materials) may be found in the online DOI or iMeta Science http://www.imeta.science/.
